# Spectral Photon-Counting CT Imaging of Gold Nanoparticle Labelled Monocytes for Detection of Atherosclerosis: A Preclinical Study

**DOI:** 10.3390/diagnostics13030499

**Published:** 2023-01-29

**Authors:** Mahdieh Moghiseh, Emily Searle, Devyani Dixit, Johoon Kim, Yuxi C. Dong, David P. Cormode, Anthony Butler, Steven P. Gieseg, MARS Bioimaging Ltd.

**Affiliations:** 1 Department of Radiology, University of Otago, Christchurch 9016, New Zealand; 2 MARS Bioimaging Ltd., Christchurch 8041, New Zealand; 3 Free Radical Biochemistry Laboratory, School of Biological Sciences, University of Canterbury, Christchurch 8041, New Zealand; 4 Department of Physics and Astronomy, University of Canterbury, Christchurch 8041, New Zealand; 5 Departments of Radiology, Bioengineering, University of Pennsylvania, Philadelphia, PA 19104, USA; 6 European Organization for Nuclear Research (CERN), 1211 Meyrin, Switzerland

**Keywords:** spectral photon-counting computed tomography (SPCCT), atherosclerosis, gold nanoparticle, imaging, labelled nanoparticles

## Abstract

A key process in the development of atherosclerotic plaques is the recruitment of monocytes into the artery wall. Using spectral photon-counting computed tomography we examine whether monocyte deposition within the artery wall of ApoE-/- mouse can be detected. Primary mouse monocytes were labelled by incubating them with 15 nm gold nanoparticles coated with 11-mercaptoundecanoic acid The monocyte uptake of the particle was confirmed by electron microscopy of the cells before injection into 6-week-old apolipoprotein E deficient (ApoE-/-) mouse that had been fed with the Western diet for 10 weeks. Four days following injection, the mouse was sacrificed and imaged using a MARS spectral photon counting computed tomography scanner with a spectral range of 7 to 120 KeV with five energy bins. Imaging analysis showed the presence of X-ray dense material within the mouse aortic arch which was consistent with the spectral characteristic of gold rather than calcium. The imaging is interpreted as showing the deposition of gold nanoparticles containing monocytes within the mouse aorta. The results of our study determined that spectral photon-counting computed tomography could provide quantitative information about gold nanoparticles labelled monocytes in voxels of 90 × 90 × 90 µm^3^. The imaging was consistent with previous micro-CT and electron microscopy of mice using the same nanoparticles. This study demonstrates that spectral photon-counting computed tomography, using a MARS small bore scanner, can detect a fundamental atherogenic process within mouse models of atherogenesis. The present study demonstrates the feasibility of spectral photon-counting computed tomography as an emerging molecular imaging modality to detect atherosclerotic disease.

## 1. Introduction

Cardiovascular disease (CVD) is a silent and progressive disease, which is difficult to detect before the onset of a clinical event, such as a stroke or myocardial infarction. The underlying pathology of CVD is the formation of atherosclerotic plaques within the artery wall [1]. Atherosclerotic plaques are made of inflammatory cells, mainly macrophages, T-cells, and smooth muscle cells, which are recruited into the inner wall of the artery [2]. Plaque progression is driven by chronic inflammatory processes which are exacerbated by a range of factors, including high plasma cholesterol, smoking, diabetes and age [3]. The growth of plaque due to the increasing number of these inflammatory cells in the artery wall reduces the artery elasticity and blood flow to key organs. In advanced plaques, significant cell death occurs leading to plaque destabilisation and rupture. The exposed plaque interior components trigger clot formation which may block the artery downstream from the ruptured plaque.

Artery plaques are difficult to detect by biochemical testing (blood or urine samples) or by imaging until they reach a significant size, at which point patients are usually experiencing various symptoms as a result of the plaque(s) presence or even its rupture [4,5,6]. Though plaques are sites of chronic inflammation, to date it has been difficult to devise definitive markers of plaque growth or destabilisation [7]. As a result, clinicians rely on the measurement of various risk factors to identify at-risk individuals for potential treatment. Artery plaques range in thickness from half a millimetre to three millimetres with a tissue composition similar to the surrounding tissues when viewed by X-ray or ultrasound imaging. In the advanced stages of the disease plaques can become calcified, so are relatively easy to detect by X-ray imaging. This makes the imaging of these plaque structures at an early or intermediate stage very challenging when calcification has not occurred. Even in advanced plaques it is difficult to detect the formation of unstable necrotic core regions by radiological imaging.

Magnetic resonance imaging (MRI), magnetic resonance angiography (MRA), and multidetector computed tomography angiography (MDCTA) are the leading imaging tools to detect aortic diseases [8]. The main challenge with MRI and MRA is a prolonged acquisition time that causes significant artefacts due to respiratory motion. This motion reduces the contrast-to-noise ratio and spatial resolution and requires ECG-gating to correct motion artefacts [9]. MDCTA uses a multirow detector to image a larger field of view, enabling shorter scan times, improved signal-to-noise ratio, and fewer image artefacts. However, difficulty in differentiating intramural hematoma, atherosclerotic plaque, and thrombus, all of which are highly attenuating, remains the primary concern in interpreting the result [10]. Although functional molecular imaging like positron emission tomography (PET)/CT or less commonly used PET/MRI can resolve this problem [11], PET availability, associated costs, low spatial resolution (about 6 mm) [12] and radionuclide radiation exposure limit the usage of these types of imaging techniques [13].

Spectral photon-counting CT (SPCCT) is a new generation of CT scanner that has received much attention in recent years [14,15,16]. SPCCT has evolved the concept of dual-energy CT (DECT) to emerge as the potential future of clinical CT imaging [14,17]. All elements have unique spectral attenuation properties. Suitable X-ray spectral characteristics within the diagnostic energy range allow key elements to be readily identified spectrally. The use of only two energy bands in DECT limits the specific identification of a number of materials, with DECT only able to separate contrast agents with significantly different X-ray attenuation coefficients. In contrast, SPCCT uses photon processing detectors, which differentiate a single broad X-ray spectrum into multiple energy bins. As a result, SPCCT is able to characterize multiple tissues, contrast agents, and even closely attenuating materials in a single acquisition with high CT contrast sensitivity and accuracy [18,19]. SPCCT has the potential to differentiate intramural hematoma from atherosclerotic plaque, even in the absence of a contrast agent [20,21,22].

Using SPCCT based MARS-CT machines developed by MARS Bioimaging Ltd., we have imaged ex-vivo human atherosclerotic plaques [23], crystalline arthropathies [24] and different types of calcium [25], excised osteoarthritic cartilage [26], high-Z materials including functionalized and non-functionalized nanoparticles [19,27], and soft tissue quantification [28] at sub-millimetre resolution.

SPCCT imaging offers the possibility to track specific cell types using nanoparticle labelling of cells [29,30]. A key early process driving plaque formation is the requirement of monocytes and macrophages into the artery wall [31]. Detection of this process has the potential to detect active plaque progression at a very early stage of the disease. In this research we examine whether SPCCT can detect the recruitment of AuNP-labelled monocytes into a mouse’s artery wall. Previous work had shown that the injection of AuNP-labelled monocytes into ApoE-/- mice resulted in the collection of X-ray dense material in the aortic arch of the mouse [32]. 

## 2. Materials and Methods

The scanner employed for this study was the MARS small animal spectral scanner, which uses a camera that incorporates seven Medipix3RX (Medipix3RX Collaboration, CERN) chips [33]. Each chip contains 128 × 128 pixels with a pixel pitch of 110 µm and is bump bonded to a cadmium zinc telluride (CZT) sensor layer. The MARS scanner features an energy resolution of 2.5 keV FWHM (full width half maximum) and can operate up to eight energy bins within the diagnostic energy range (20–120 keV). The MARS system enables simultaneous identification and quantification of multiple materials through individual photon energy measurements [34].

Data was acquired using a micro-focus poly-energetic X-ray source with a tungsten target (SB-120-350; Source-Ray Inc, Ronkonkoma, NY, USA). The energy thresholds were chosen to take into account the gold K-edge (80.7 keV) while retaining high resolution of bone and soft-tissue. To prevent imaging artifacts caused by narrow energy bins, a minimum of 10 keV intervals was used between thresholds. Based on the test with phantoms, the energy bins of 7–40, 40–50, 50–60, 60–79, and 79–118 keV were selected as they gave good differentiation between gold nanoparticles (AuNPs), calcium, and soft tissue in the spectral response. Reconstruction of the acquired data was performed using an in-house algorithm, referred to as MARS-recon [34]. The experimental setup is summarised in Table 1.

Pixel masking was applied prior to scanning to remove noisy pixels, including non-functional, high and low sensitive pixels [35]. The raw data was collected into DICOM (Digital Imaging and Communications in Medicine) files and transferred to the scanner’s image processing system. The acquired data from five energy bins was flatfield corrected and ring artefacts filtered before MARS_recon (iterative reconstruction algorithm [36]) simultaneously reconstructed all energy bins into an 834 × 834 pixels, using a voxel size of 90 × 90 × 90 µm^3^. 

Two material decomposition (MD) methods were applied. Both methods required a material calibration phantom. The first method used the calibration phantom spectral response as a reference. The spectral response of individual pixels at the region of interest within the biological sample (aorta) were compared to the reference to determine what material was present and at what concentration. A semi-automatic program was employed to process each pixel and measure the spectral response. The second method employed in-house MD software (MARS MD), which uses the reconstructed attenuation images from all energy bins to produce material density images for a set of preselected materials. Each material needs a set of mass attenuation coefficients to relate attenuation to density. These were obtained experimentally using the material calibration phantom. The MD algorithm is based on a non-negative constrained linear least-squares technique [34,37]. Both MD methods were applied to the same mouse dataset.

### 2.1. Material Calibration Phantom

**Figure 1:**
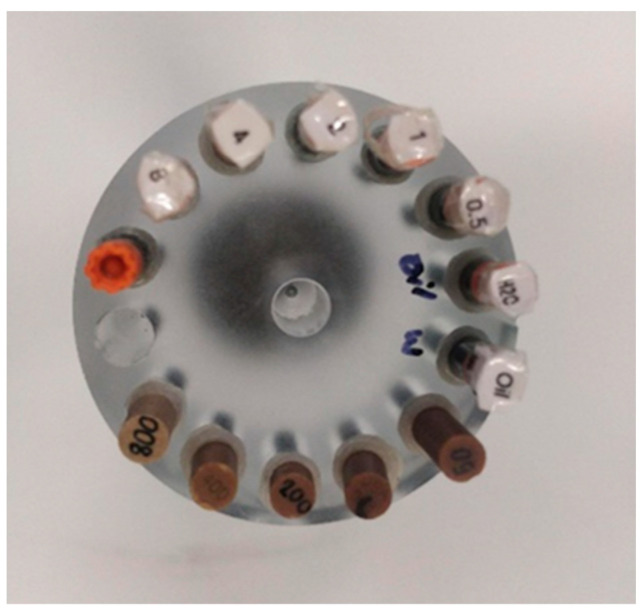
Photo of the calibration phantom. PMMA calibration phantom with eight 200µL Eppendorf PCR inserts and five HA rod inserts.

The reference spectral responses and effective mass attenuation coefficients needed for MD were obtained by scanning a calibration phantom. A customized PMMA (polymethyl methacrylate) phantom held 200 µL Eppendorf PCR tubes containing AuNP solutions of various concentrations (0.5, 1, 2, 4, 8 and 10 mg/mL), hydroxyapatite (HA) rods (HA 50, 100, 200, 400 and 800 mg/cm^3^; HA acquired from QRM GmbH), water, and lipid (Figure 1). To evaluate the effect of soft tissue on the material attenuation profile, a layer of lamb red meat was wrapped around the phantom. The calibration phantoms were used to evaluate the linearity of the response to AuNP and HA concentrations and evaluate the spectroscopic response of the detector for different materials.

The same scanning protocol was used to image a material calibration phantom (Figure 1) to provide reference spectral responses and effective mass attenuation coefficients. The reconstructed spectral response images of the phantom were analysed [38]. Linear regression analysis determined the relationship between spectral response and material concentration (linearity response) [39]. Furthermore, the relationship between the spectral response and the energy thresholds was evaluated graphically (spectral response). 

### 2.2. AuNPs Synthesis and Characterization

The 11-mercaptoundecanoic acid (11-MUDA) coated gold nanoparticles used in this study had a diameter of 15 nm and were synthesized from gold (III) chloride hydrate using the Turkevich method as previously described [32]. In brief, 85 mg of the gold salt were dissolved in 250 mL of deionized water and brought to a boil. 25 mL of sodium citrate (38.8 mM) were added, the solution boiled for 15 min, and then cooled to room temperature. The particles were coated in 11-MUDA by ligand exchange in ethanol before washing in water by centrifugation and sterilization through a 0.45 μm syringe filter [32]. 11-MUDA was selected on the basis of previous studies, which screened NP coated with several different ligands. 11-MUDA coated NP were found to result in high nanoparticle uptake without adversely affecting cell viability or function. An inductively coupled plasma optical emission spectrometer (ICP-OES) was used to measure AuNP uptake. The concentrations varied somewhat depending on the batch, but were typically about 100 mg/mL. Using dynamic light scattering (DLS) and zeta potential, the particles were determined to have a hydrodynamic diameter of 31.5 nm and a surface charge of −36.8 eV.

### 2.3. Mouse Preparation

Six-week-old ApoE-/- mouse (B6.129P2-ApoEtm1UnC/J) was fed with the Western diet (Research Diets Inc, New Brunswick, NJ) for 10 weeks to develop atherosclerosis. Primary monocytes from C57BL/6 mice were treated with 0.5 mg/mL 11-MUDA AuNP. After 24 h cells were washed two times with DPBS, and then were resuspended in 250 μL of DPBS. A dose of 1.0 × 10^6^ cells were injected intravenously into each mouse. The mouse was euthanized using CO_2_ 5 days after injection and scanned with MARS SPCCT. Following that, aortic plaques were excised, and transmission electron microscopy (TEM) was used to confirm the presence of gold-labelled monocytes. The animal experiment was performed at the University of Pennsylvania. Animal ethics for this study were approved by University Laboratory Animal Resources in conjunction with the Institutional Animal Care and Use Committee at the University of Pennsylvania.

## 3. Results

### 3.1. Phantom Imaging

**Figure 2:**
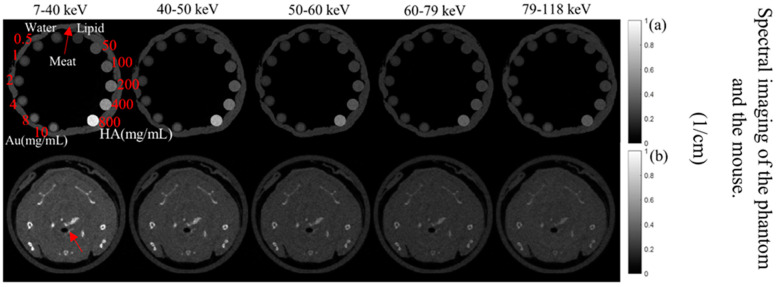
Axials slices of the calibration phantom and mouse data. (**a**) Reconstruction of the calibration phantom for each energy bin (as indicated). Phantom contains Au, HA, lipid, and water. (**b**) Axial view of energy images of the mouse-the red arrow indicates the aortic arch.

**Figure 3:**
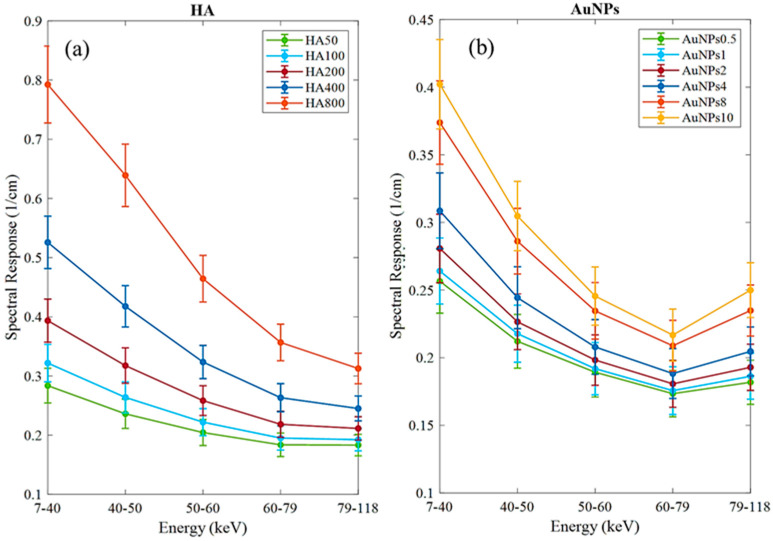
Detector spectral response. (**a**) Spectral response of the detector for HA calibration rods. (**b**) Spectral response of the detector for AuNPs vials. Attenuation enhancement is observed in energy bin 5 (79–118 keV), indicating the K-edge of Au; 80.7 keV.

**Figure 4:**
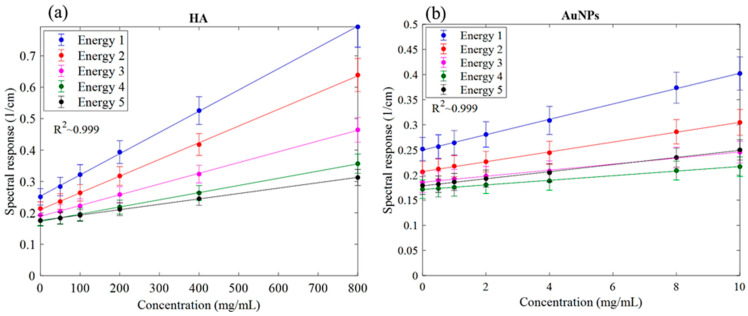
Linearity of the detector spectral response. (**a**) Linearity of attenuation of HA (Spectral response). (**b**) The linearity of attenuation of AuNPs (Spectral response). The result of the linear regression shows that the CT system is linear with R^2^ values indicated on the graph. Accuracy of material identification and decomposition is based on the linearity established for each energy bin, using the calibration vials.

The MARS imaging system was used to detect four different materials, AuNPs, water (soft tissue), lipid, and hydroxyapatite (HA) rods (bone). In Figure 2a, a series of selected slices in each energy bin of the calibration phantom is shown. The spectral response is plotted in Figure 3a,b for the HA and AuNPs concentrations, respectively. As it can be seen in Figure 3, HA and AuNPs have different spectral response trends, due to their different attenuation profiles. The spectral response of HA decreases as the energy thresholds increase, whereas the spectral response of the AuNP concentrations decreases until the fourth energy threshold, and increases in the fifth energy threshold to represent the k-edge absorption of gold at 80.7 keV. The linearity responses for AuNP and HA are plotted in Figure 4a,b. The two different trends were considered as the references in the first MD method to detect AuNPs in the aortic region. Establishing linearity of attenuation for each energy bin results in accurate material quantification and plays a key role in the result interpretation. The spectral response for AuNPs increases at the fifth threshold energy, whereas spectral response for HA decreases with increasing energy thresholds.

**Figure 5:**
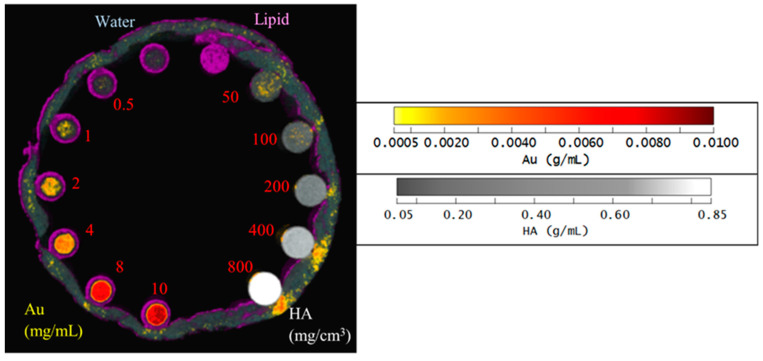
Material image of phantom. 3D rendering material image of the phantom; lipid (purple), AuNPs (yellow to red), HA (grey to white) and water (blue).

Figure 5 shows the material image of the phantom using the second MD method. HA vials at a concentration of 200 mg/cm^3^ and above identify correctly as HA by 100%, while for the lower concentrations of HA 100 and 50 mg/cm^3^, about 5% and 15% are misidentified as gold at about 8 mg/mL, respectively. For AuNP vials at concentrations 10, 8 and 4 mg/mL, the correct identification is above 97%. However, for lower concentrations of AuNPs, the correct identification was about 40 to 75%. Correct identification of 80% or more is acceptable or considered reliable [19]. Soft tissue, when near higher concentrations of HA were often misidentified as AuNP due to beam hardening caused by HA at higher concentrations.

### 3.2. Mouse Imaging

**Figure 6:**
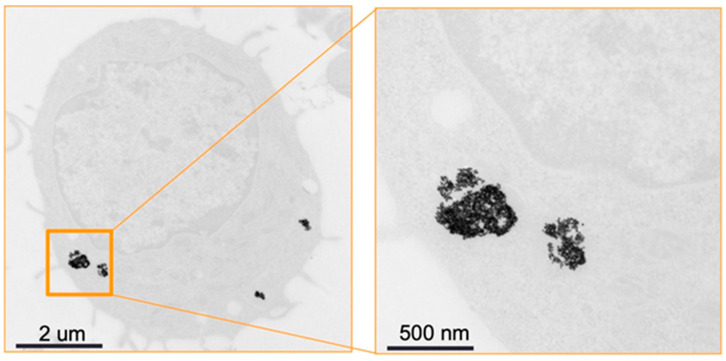
TEM image. TEM of monocytes incubated with 11-MUDA coated gold nanoparticles. The panel on the right is an enlargement of the boxed area in the panel on the left.

Figure 6 shows TEM of a primary monocyte incubated with 11-MUDA coated gold nanoparticles. The uptake for each cell was 127 pg/cell, as measured by ICP-OES.

**Figure 7:**
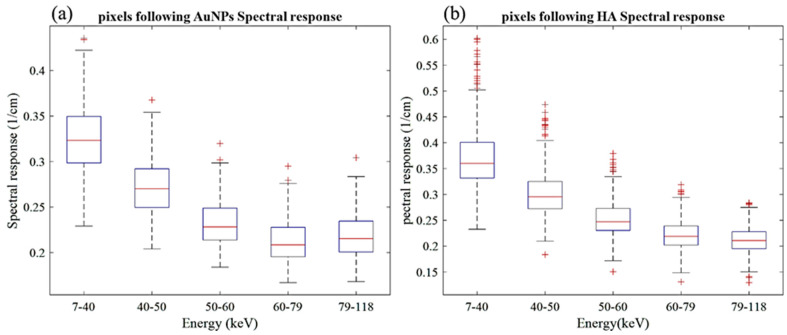
Spectral response of voxels located within atherosclerotic regions. (**a**) Voxel response follow the AuNP trend peak at the fifth energy bin. (**b**) Voxels’ spectral responses follow HA attenuation profile.

**Figure 8:**
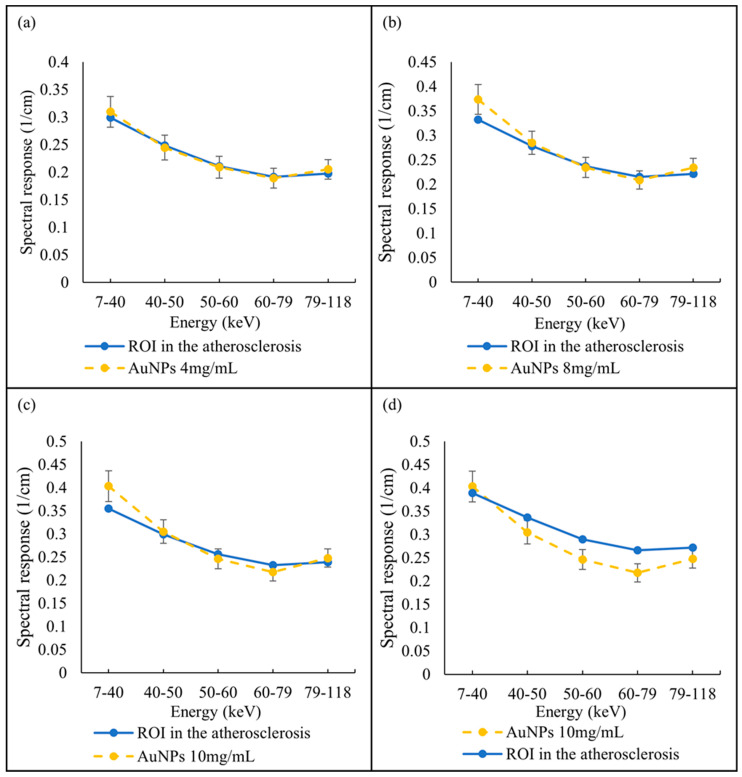
Quantify AuNPs located within atherosclerotic regions. Spectral response of voxels located within atherosclerotic regions follow the AuNPs trend pick at fifth energy bin and quantified as (**a**) 4 mg/mL, (**b**) 8 mg/mL, (**c**) 10 mg/mL, and (**d**) more than 10 mg/mL, in comparison with known AuNP concentrations within phantom.

A selected slice in each energy bin for the site of interest in the mouse (aortic arch) is shown in Figure 2b. The aortic arch was the region of interest (ROI) within the ApoE-/- mouse where atherosclerosis had formed. Therefore, this region was suspected to recruit AuNP-labelled monocytes. To apply the first MD method on the mouse data, the ROIs were selected in the aortic arch along 10 slices to cover the volume in question. In total, 890 voxels were selected. The spectral response was analysed for all selected voxels. The attenuation threshold for soft tissue is also set, and therefore the voxels whose attenuation is higher than the threshold are considered to contain AuNPs or calcification. The results showed 207 voxels in an aortic arch followed the spectral response of AuNP, which indicated a peak in the fifth energy bin, while the remaining voxels reflected the HA spectral response (Figure 7). To measure the concentration of gold within the 207 voxels exhibiting an AuNP spectral response, the voxel attenuation profile was compared across all energy bins with the attenuation profiles of known concentrations of AuNPs in the calibration phantom. The data show that out of 207 voxels, 96, 57, and 20 voxels were quantified to be 4, 8, and 10 mg/mL of AuNPs, respectively, with 33 voxels indicating a concentration more than 10 mg/mL (Figure 8).

**Figure 9:**
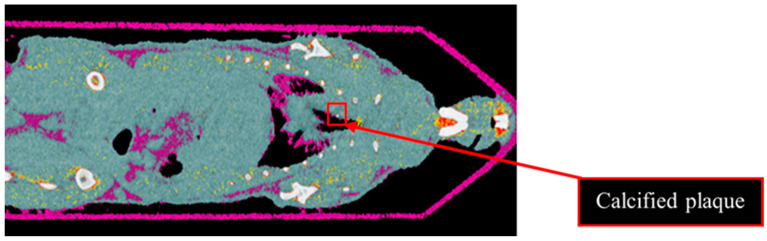
Material image of ApoE-/- mouse. The 3D material image of ApoE-/- mouse. Bone (white), AuNPs (yellow to red), soft tissue (blue) and lipid (purple). Ring artefact is misidentified as AuNPs. The red box indicates calcified plaque.

**Figure 10:**
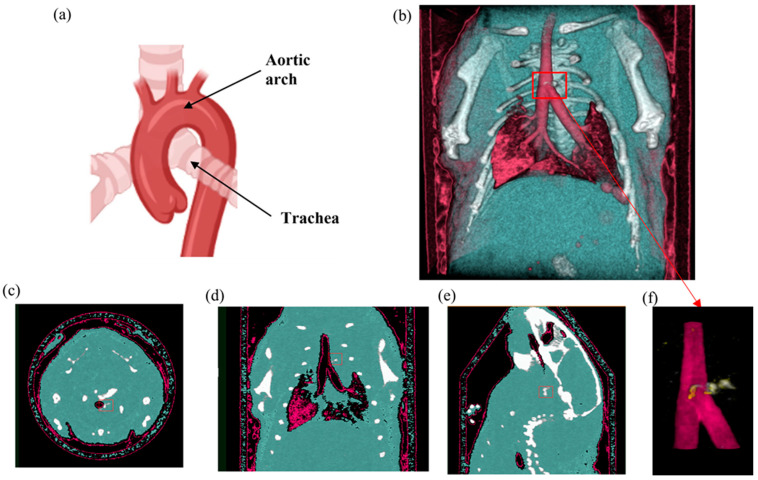
Mouse SPCCT imaging. (**a**) Mouse heart and aortic arch anatomy. (**b**) MARS 3D visualisation of fused MD images including energy image at E1 (pink), HA channel (white), and soft tissue (blue). (**c**–**e**) Axial, coronal and sagittal slices through the AuNP ROI in the aorta respectively. (**f**) MARS fused MD images of the site of the interest (atherosclerotic plaque) including energy image at E1 (pink), HA channel (white) and yellow indicating ROI containing AuNPs determined by using first MD method. The red box indicates the aortic arch.

Results from the second MD method show the four fused material channels, consisting of AuNP, HA, water and lipid (Figure 9). Due to a ring artefact, AuNP is observed throughout the mouse. Considering the result of the second MD method on the phantom, AuNPs at a concentration of 4 mg/mL and lower can be misidentified as HA, and consequently, AuNPs within plaque are very likely to be misidentified as HA. Moreover, as can be seen in Figure 5, beam hardening caused by high concentrations of HA caused the soft tissue near the HA rods to be misidentified as gold. Therefore, the material density images produced by the second MD method do not provide sufficient evidence of the presence of AuNPs within the aortic plaque. However, the first MD method confirmed the presence of AuNPs in the aortic plaque. We therefore fused one energy channel (E1) (lung and trachea), HA (bone using second MD method), and water (soft tissue), and AuNPs (using first MD method) to indicate AuNPs in the plaque. In Figure 10a the anatomy of the heart and aortic arch can be seen for comparison. A MARS-CT image indicating AuNPs in the aortic region with a high level of detail, including the aortic arch shape is shown in Figure 10.

**Figure 11:**
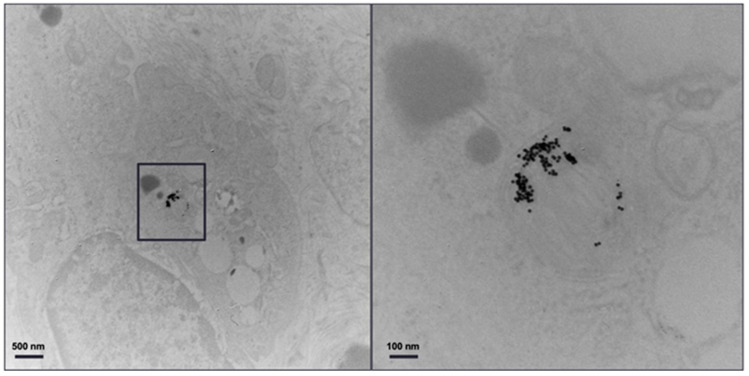
TEM of plaque. Transmission electron microscopy of the atherosclerotic plaque of ApoE-/- mouse injected with gold nanoparticle labelled monocytes. The panel on the right is an enlargement of the boxed area on the left.

Figure 11 shows the TEM image of the excised plaque that confirms the presence of AuNPs. Note that control groups of mice (i.e., mice without atherosclerosis injected with AuNP labelled monocytes and mice with atherosclerosis injected with unlabelled monocytes) were scanned with conventional CT, as previously published [32], where the specific imaging of AuNP labelled monocytes in atherosclerosis was proven.

## 4. Discussion

Using MARS-CT, SPCCT imaging was able to identify and quantify the recruitment of gold-labelled monocytes in an ApoE-/- mouse. The findings were in agreement with the results obtained by micro-CT and electron microscopy using the same nanoparticles [32]. The SPCCT imaging using a single multi energy scan confirmed the high attenuating material in the aortic arch followed the spectral response of gold. Specifically, MARS-CT was able to differentiate AuNPs from calcification (hydroxyapatite). Here we have shown that the SPCCT can directly identify and quantify AuNP-labelled monocytes by the unique spectrum of the gold nanoparticles measured by the MARS-CT scanner.

Previous analysis using micro-CT required scanning of the mice before and after injection to calculate changes in attenuation to confirm the presence of gold labelled monocytes [32]. That analysis showed a 15.3 HU increase in the aortic arch region at day 5 in comparison to day 0 (before injection). In the previous study the presence of gold in the artery was confirmed by destructive ICP-OES analysis. With SPCCT imaging, tissue destruction was not necessary to confirm the presence of gold, as this was confirmed by the recorded X-ray adsorption. In addition to confirming the presence of gold, the MARS-CT was able to quantify the different concentrations of the materials within the scanned regions. Figure 2 shows how the spectral response of AuNP is distinct from HA. The linearity of attenuation for each energy bin in Figure 4a,b represents the capability of MARS SPCCT to identify and quantify HA and AuNPs. In spite of the fact that the collected spectral response for each material was significantly different from the other material, the first MD algorithm using least square error was not successful in differentiating AuNP-labelled monocytes from calcification within an aortic arch. However, the second MD method, which has examined only spectral response, was capable of distinguishing AuNPs-labelled monocytes from calcified regions within an aorta. In the last decade, many studies have focused on the structure of atherosclerotic plaques, and the resulting clinical outcomes [40,41]. To predict the risk of plaque rupture and the resulting clinical events, it is important to determine the plaque characteristics. Microcalcification and inflammation within the plaque have been evaluated as some of the causes of plaque rupture and cap erosion [42,43], with some studies suggesting macrocalcification may indicate increasing plaque stabilisation [44]. Inflammation drives the recruitment of monocytes into the artery wall, leading to plaque growth and instability [45,46]. Several studies have been carried out to explore the potential of MARS SPCCT to characterize atherosclerotic plaque. In studies conducted by Searle et.al [21] and Dahal [20], the SPCCT has been successfully used to locate and identify iron deposits and calcium within atherosclerotic plaque, both of which are considered critical biomarkers of vulnerable plaque. In another pilot study we investigated the composition of late-stage vulnerable plaques in ApoE mice fed on a chow diet. These mice (over 12 months old) show calcifications in the aortic arch, ascending aorta, and aortic root. The current work shows that imaging AuNPs labelled monocytes using SPCCT has the potential to characterize plaque.

SPCCT imaging has also been successfully used to image and differentiate cells from their embedding scaffold in brain tissue [47]. This imaging required the use of two different contrast agents which were easily distinguished from each other by their different X-ray absorption spectrum by SPCCT imaging, showing the strength of spectral imaging, especially in animal disease models using nanoparticle contrast agents.

The development of molecular imaging of specifically targeted cells or biomolecules within tissues has a great capacity for distinguishing characteristics of atherosclerotic plaque. There are several hurdles to overcome, such as radiation exposure [9], motion artefact [48], and spatial resolution [49], which limit imaging of arteries, especially coronary arteries. PET and single-photon emission computerized tomography (SPECT) do overcome some of these problems, but availability and requirement of the use of radio-labelled pharmaceuticals are two major limitations which restrict the use of these techniques for diseases like atherosclerotic plaque, which require follow-up imaging [50]. The other limitation of PET and SPECT is that they cannot provide anatomical information without being combined with CT or MRI. 

It is anticipated that SPCCT scan time will be reduced with further with sensor development, so reducing motion artifacts, as seen with conventional CT.

MARS SPCCT can overcome other issues due to its high spatial resolution, low radiation dose, and the absence of needing radionuclide imaging agents. The results described herein were obtained from scanning the mouse post-mortem, which therefore eliminated any issues that could be created by subject motion. In vivo scanning would benefit from respiratory gating approaches such as algorithms applied to the reconstructed images to minimize motion artefacts. 

The current study has a number of limitations. First, we have only studied one type of photon counting scanner, therefore it is not clear how generalizable the results are to the different types of systems in development (although data on macrophage imaging with AuNP in atherosclerotic plaque using a different scanner are promising [51]). Second, we focused on the aortic arch, which is where plaque first develops in this early model of atherosclerosis in mice. However, screening the whole mouse body did not indicate any calcified or uptake of AuNPs-labelled monocytes. Future studies might examine other arteries in other types of models of atherosclerosis as monocyte recruitment into plaques is a key feature of plaque development. Moreover, the currently used system is designed for small animal imaging, and results may differ with clinical scale scanners. From a dose perspective, this approach would be feasible in humans, since only approximately 1 g of AuNP would be needed, which is affordable. A key question for clinical translation is the safety and excretion of the nanoparticles. To achieve this, the latter, smaller, renally clearable AuNP may need to be employed. In addition, the results described herein were obtained from scanning the mouse post-mortem, which therefore eliminated any issues that could be created by subject motion. In vivo scanning may be possible via respiratory gating approaches such as algorithms applied to the reconstructed images to minimize motion artefacts.

## 5. Conclusions

We have demonstrated the feasibility of SPCCT to detect active atherosclerotic disease in conjunction with nanoparticle labelling of cells. SPCCT imaging using the MARS-CT was capable of distinguishing AuNP labelled monocytes from calcified regions within the atherosclerotic plaque based on the X-ray absorbance spectrum of gold. This imaging procedure could be used to study the kinetics of monocyte requirement during plaque development in animal models and demonstrates the feasibility of using targeted nanoparticles for clinical diagnosis.

## Figures and Tables

**Table 1:** Summary of MARS spectral CT scanning protocol. SDD: source-to-detector distance. SOD: source-to-object distance.

Scan Type	Helical Scan
Tube voltage	118 kVp
Tube current	34 µA
Exposure time	220 ms
SDD, SOD	276.95 mm, 211.95 mm
Field of view	60 mm
Circular projections, Flat fields	720, 720
Voxel size	90 × 90 × 90 µm^3^
Filtration	1.96 mm Al
Energy bins	7, 40, 50, 60, 79 keV
Slice thickness	90 µm
Pixel pitch	110 µm
Collimation width	125 µm

## Data Availability

We have uploaded the whole mouse imaging to the public repository, Available online: https://figshare.com/s/d77f95e3ff3f661c2cac and DOI: https://doi.org/10.6084/m9.figshare.19661391 (accessed on 27 April 2022).
